# Possible role of l‐carnitine in improvement of metabolic and hepatic changes in hyperuricemic and hyperuricemic—Fructose‐supplemented rats

**DOI:** 10.14814/phy2.14282

**Published:** 2019-11-28

**Authors:** Bataa M. A. El‐Kafoury, Mona A. Ahmed, Gehad A. Hammouda, Amr H. ElKady, Noha N. Lasheen

**Affiliations:** ^1^ Physiology Faculty of Medicine Ain Shams University Cairo Egypt; ^2^ Histology and Cell Biology Faculty of Medicine Ain Shams University Cairo Egypt

**Keywords:** fructose, hyperuricemia, l‐carnitine, liver, metabolic, oxidative stress

## Abstract

Hyperuricemia was linked to diabetes mellitus, metabolic syndrome, and oxidative stress, and could be induced by higher fructose consumption through altering energy status in liver. l‐Carnitine is an antioxidant, affecting mitochondria and cellular energetics; however, little is known about its effects in hyperuricemic states. This study investigated metabolic and hepatic effects of hyperuricemia and fructose feeding, and demonstrated the role of l‐Carnitine in such states. Fifty adult male Wistar rats were randomly divided into control, untreated hyperuricemic, fructose‐supplemented hyperuricemic, l‐Carnitine‐treated hyperuricemic, and l‐Carnitine‐treated fructose‐supplemented hyperuricemic groups. The separated plasma was used for determination of the glycemic control, lipid profile, liver function tests, uric acid level, and oxidative stress markers. Atherogenic index, HOMA‐IR, and body mass index (BMI) were calculated. Left liver lobe and left kidney specimen from all groups were used for histopathological studies. Hyperuricemic rats exhibited significantly hypoalbuminemia, dyslipidemia, insulin resistance, and oxidative stress compared to the controls. Fructose‐supplemented hyperuricemic group showed obesity and more deleterious effects, as well as, steatosis, and renal tubular damage compared to the hyperuricemic rats. Concomitant l‐Carnitine treatment with hyperuricemia improved such effects, despite causing adiposity. While combined l‐Carnitine treatment and fructose supplementation in hyperuricemia limited the aggressive hyperuricemic picture of fructose supplementation. It is concluded that hyperuricemia has detrimental metabolic and hepatic effects. Artificial fructose supplementation worsened such effects, while l‐Carnitine was efficient in ameliorating these hyperuricemia and/or excess fructose‐induced hyperuricemia effects, through its anti‐inflammatory, antisteatotic, and antioxidant properties.

## Abstract 2

Hyperuricemia has detrimental metabolic and hepatic effects. Artificial fructose supplementation worsened such effects, while l‐Carnitine was efficient in ameliorating these hyperuricemia and/or excess fructose‐induced hyperuricemia effects, through its anti‐inflammatory, antisteatotic, and antioxidant properties.

## Graphical abstract

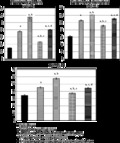

## 1 INTRODUCTION

Hyperuricemia, the deposition of urate crystals in the joints caused by high uric acid level in the blood (Wu et al., 2014), could result from either overproduction of uric acid (10% of hyperuricemic cases), or from lowered uric acid excretion (90% of cases), or both (Wolff, Cruz, Vanderman, & Brown, 2015). Hyperuricemia in adults could ensue when blood uric acid is more than 7 mg/dl in men and 6 mg/dl in women. This sex difference was linked to the uricosuric effect of estrogens in women (Mumford et al., 2013).

On the other hand, normal uric acid level was found to be lower in mammals, such as rats and mice, than in humans because uric acid might be further oxidized to allantoin by uricase in mammals (Desideri et al., 2014). However, the gene expressing uricase enzyme could be mutant in human, thus uric acid could be the final end product of both endogenous and exogenous purine catabolism (Mandal & Mount, 2015).

Also, many studies reported that hyperuricemia could be associated with abdominal obesity in males and females (Silva, Carraro, Bressan, & Hermsdorff, 2015) and many metabolic disorders such as nonalcoholic fatty liver disease (NAFLD), type 2 diabetes mellitus, obesity, and hypertension (Li et al., 2015). It was found that metabolic syndrome could be affected by uric acid level (Srikanthan, Feyh, Visweshwar, Shapiro, & Sodhi, 2016). Also, Baldwin et al. (2011) mentioned that hyperuricemia could induce the pro‐inflammatory endocrine imbalance, particularly at adipose tissue, inducing insulin resistance. Thus, serum uric acid was found to be higher in metabolic syndrome states, elevating the number of components of the metabolic syndrome (Silva et al., 2015).

In addition, Kang, Park, Lee, and Johnson (2005) reported that uric acid, in mature adipocytes, could initiate nicotinamide adenine dinucleotide phosphate (NADP) oxidase activity, thereby producing reactive oxygen species (ROS) (Sautin, Nakagawa, Zharikov, & Johnson, 2007). In a later study, hyperuricemic rats had prominent oxidative stress resulting in hepatocyte damage (Lanaspa et al., 2012). Therefore, Billiet, Doaty, Katz, and Velasquez, (2014) reported that uric acid could be a marker for oxidative damage in many conditions such as atherosclerosis and diabetes. On the contrary, uric acid could be responsible for 2/3 of total plasma antioxidant capacity due to its double bonds (Sautin & Johnson, 2008), and could directly inhibit ROS such as peroxyl radical and peroxynitrite, protecting the cell membrane and DNA (de Oliveira & Burini, 2012).

On the other hand, Essawy, Abdel‐Sater, and Elbaz (2014) demonstrated that fructose could be the causal factor of metabolic syndrome and obesity due to its ability to elevate uric acid. Controversial studies are present describing the relation between fructose consumption and uric acid level, which was found to be transient rise in normal person (Carran, White, Reynolds, Haszard, & Venn, 2016), or limited to gouty subjects (Menghini & Della Corte, 1987), or even no association (Wang et al., 2012). Thus, it is of value to demonstrate the effects of fructose consumption on uric acid in normal states in this study.

l‐Carnitine (4‐N‐trimethyl ammonium 3‐hydroxybutyric acid), the biologically active stereoisomer of carnitine, is supplied exogenously through meat ingestion, and can be synthesized endogenously in the liver, kidney, and brain from the amino acids l‐lysine and l‐methionine (Sakai et al., 2016). l‐Carnitine supplementation was found to be beneficial in treating obesity and improving glucose intolerance and total energy expenditure (Flanagan, Simmons, Vehige, Willcox, & Garrett, 2010). Also, it has antisteatotic and hypolipidemic effects on liver metabolism (Kolodziejczyk, Saluk‐Juszczak, & Wachowicz, 2011), protecting plasma components against oxidative stress (Ribas, Vargas, & Wajner, 2014).

In addition, it was found that the uric acid production could be prevented by l‐Carnitine administration (Loots, Mienie, Bergh, & Schyf, 2004). However, no recent studies were found to investigate the effects of l‐Carnitine on fully developed state of hyperuricemia. Also, the interaction of l‐Carnitine supplementation and fructose supplementation in hyperuricemia was not clearly demonstrated yet, it is of value to study whether l‐Carnitine affects the state of hyperuricemia with or without fructose supplementation, or it could limit other hyperuricemic effects.

### 1.1 Aim of the work

This study compared hyperuricemia alone and combined hyperuricemia with fructose feeding on the possible metabolic and hepatic effects in adult male rats, shedding more light on histopathological changes in liver and kidneys. Also, it tried to probe the ability of l‐Carnitine to manage such effects of hyperuricemia.

## 2 MATERIALS AND METHODS

This study was performed on 50 adult male Wistar rats, initially weighing 150–180 g, purchased from Ophthalmic Diseases Research Institute, Giza, and were housed in the Physiology Department Animal House, Faculty of Medicine, Ain Shams University under standard conditions of boarding at room temperature 22–25°C, 12 hr light dark cycle, and free access to food and water—ad libitum—throughout the whole period of the study. Standard rat diet was introduced daily at fixed time to the rats. All animal experiments were performed according to the National Institutes of Health guide for the care and use of Laboratory animals (NIH Publications No. 8023, revised 1978). At the end of experiment, animals were killed by overdose of anesthesia. Animal remains disposal occurred by incineration.

### 2.1 Chemicals and drugs

The used chemicals were oxonic acid potassium salt (Alfa Aesar Co, Germany), l‐Carnitine 1g/5 ml ampule (Mepaco pharmaceuticals, Egypt), and fructose powder (Uni Fructose, from Uni Pharma, Egypt).

### 2.2 Experimental protocol

Rats were randomly divided into the following groups (each group consisted of 10 rats):

Group I: Control group: they were i.p. injected with normal saline solution in an equivalent volume to that in which oxonic acid potassium salt was dissolved.
Group II: Untreated Hyperuricemic group: which was i.p. injected with oxonic acid potassium salt (250 mg kg^−1^ day^−1^ dissolved in normal saline solution) for 4 weeks (Haidari, Rashidi, & Mohammad‐Shahi, 2012).
Group III: Untreated Hyperuricemic Fructose‐supplemented group: hyperuricemia was induced similar to group II. One hour after injection of oxonic acid potassium salt, fructose was supplemented by gavage (8 mg kg^−1^ day^−1^ dissolved in distilled water) for 4 weeks (Barbosa, Albuquerque, Faria, Oliveira, & Castilho, 2007).
Group IV: l‐Carnitine‐treated Hyperuricemic group: which was manipulated as in group II. Two hours after oxonic acid injection, l‐ Carnitine was i.p. injected (500 mg kg^−1^ day^−1^) for 4 weeks (Uysal, Yalaz, Acikgoz, Gonenc, & Kayatekin, 2005).
Group V: l‐Carnitine‐treated Hyperuricemic Fructose‐supplemented group: hyperuricemia was induced similar to group II. Fructose was supplemented by gavage (8 mg kg^−1^ day^−1^) 1 hr after oxonic acid injection, and after another 1 hr, l‐ Carnitine was supplemented to group IV.

On the day of sacrifice, fasting blood glucose (FBG) level was determined in a blood drop taken from rat tail, using one touch apparatus (All medicus Co., LTD). The overnight fasted rats were weighed and anesthetized by i.p. injection of Pentobarbitone (40 mg/kg B.W.). When the stage of surgical anesthesia was reached, naso‐anal length and waist circumference were measured.

Thereafter, the separated plasma samples after abdominal aorta cannulation were stored at −80°C for later determination of plasma levels of uric acid, insulin, nitrite (an indicator of nitric oxide), malondialdehyde (MDA), and total antioxidant capacity (TAC), in addition to lipid profile and liver function tests (γ‐GT activity and levels of albumin and ALT), using commercially available colorimetric kits. All assays were performed according to the manufacturer's instructions. Also, left kidney and left liver lobe specimen were fixed in 10% formalin for histopathological examination.

Final body mass index (BMI) was calculated according to Bernardis (1970) as follows:

$$\text{BMI}\;=\;\text{Body weight}\;\left(g\right)/{\left(\text{Naso}\;-\;\text{anal length in cm}\right)}^{2}$$

Plasma LDL‐C level was calculated according to Friedewald, Levy, and Fredrickson (1972) as follows:

$$\text{LDL}\;-\;C\left(\text{mg/dl}\right)=\text{total cholesterol}-\left[\text{HDL - C + TG/5}\right].$$

The atherogenic index was calculated according to Grundy et al. (1987) as follows:

Atherogenic index=Total cholesterol/HDL-C.

Insulin resistance was calculated by the homeostasis model assessment score (HOMA‐IR) according to Salgado et al. (2010), and by homeostatic model assessment of beta‐cell function (HOMA‐B) according to Matthews et al. (1985) as follows:

$$\begin{array}{cc} \text{HOMA-IR} & =[\text{fasting plasma insulin}\;(\mu \text{IU}/\text{ml})\;\\ & \;\times \;\text{fasting plasma glucose}\;(\text{mg}/\text{dl})/405]. \end{array}$$

HOMA-B=360∗[Fasting Insulin]/([Fasting Glucose]-63)%

### 2.3 Histopathological examination

Two cubic millimeter, cut from different areas of the renal cortex and liver tissues was fixed in 10% formalin solution, and then they were processed, embedded to obtain paraffin blocks, and cut at 5‐µm thickness sections. The sections were deparaffinized in xylol solution then rehydrated in 100%, 95%, and 70% alcohol and washed in distilled water. Then they were stained with hematoxylin and eosin stain for routine histological examination (Bancroft & Gamble, 2002).

### 2.4 Statistical analysis

All results in the present study were expressed as mean ± *SE* of the mean. Statistical package for the Social Sciences (SPSS, Inc.) program, version 20.0, was used to compare significance between each of the two groups. One‐way ANOVA for the difference between means of the different groups was performed in this study, using post hoc test. Differences were considered significant when *p* ≤ .05.

## 3 RESULTS

Hyperuricemic group showed insignificant changes in both final BMI and waist circumference compared to the control group; however, these parameters were significantly higher in concomitant fructose supplementation and hyperuricemia group and l‐Carnitine treatment to hyperuricemic rats compared to either control ones or hyperuricemic rats (*p* < .001 for each one). Meanwhile, they were significantly reduced in l‐Carnitine‐treated fructose‐supplemented hyperuricemic rats (group V) compared to its respective untreated rats (group III) (*p* < .05, *p* < .01, respectively). Although l‐Carnitine‐treated fructose‐supplemented hyperuricemic rats had significant rise in final BMI when compared to the hyperuricemic rats (*p* < .05), also, these rats had significantly elevated waist circumference compared to the control ones (*p* < .01), as shown in Table 1.

**Table 1:** Changes in final body mass index, waist circumference, plasma insulin (μIU/mL) level, and HOMA‐B in the different studied groups

	Control group (I)	Untreated		l‐Carnitine‐treated	
		Hyperuricemic group (II)	Hyperuricemic Fructose‐supplemented group (III)	Hyperuricemic group (IV)	Hyperuricemic Fructose‐supplemented group (V)
Final body mass index	0.491 ± 0.014	0.489 ± 0.007	0.550^a^, ^b^ ± 0.007	0.569^a^, ^b^ ± 0.008	0.518^b^, ^c^, ^d^ ± 0.01
Waist Circumference (cm)	12.85 ± 0.2	13.25 ± 0.13	14.2^a^, ^b^ ± 0.17	14.2^a^, ^b^ ± 0.17	13.5^a^, ^c^, ^d^ ± 0.15
Plasma Insulin Level (μIU/ml)	4.55 ± 0.08	4.7 ± 0.07	4.77 ± 0.08	4.57 ± 0.09	4.68 ± 0.08
HOMA‐B	17,000 ± 1,194	4,026.53^a^ ± 196.7	2,895^a^ ± 83.84	6,136.75^a^ ± 535.91	3,619.05^a^ ± 197.02

Note

Number of rats in each group is 10 rats.

a Significance by LSD at *p* < .05 compared to control group (I).

b Significance by LSD at *p* < .05 compared to untreated hyperuricemic group (II).

c Significance by LSD at *p* < .05 compared to untreated hyperuricemic fructose‐supplemented group (III).

d Significance by LSD at *p* < .05 compared to l‐Carnitine‐treated hyperuricemic group (IV).

**Figure 1:**
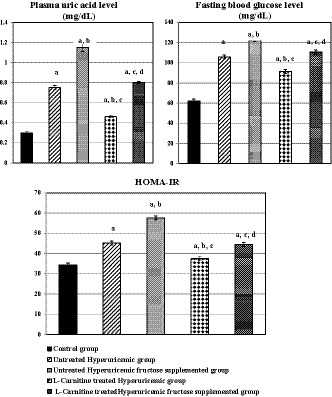
Changes in plasma uric acid level (mg/dl), fasting blood glucose levels (mg/dl), and HOMA‐IR in the different studied groups. Number of rats in each group is 10 rats. (a) Significance by LSD at *p* < .05 compared to control group (I). (b) Significance by LSD at *p* < .05 compared to untreated hyperuricemic group (II). (c) Significance by LSD at *p* < .05 compared to untreated hyperuricemic fructose‐supplemented group (III). (d) Significance by LSD at *p* < 0.05 compared to l‐Carnitine‐treated hyperuricemic group (IV)

As shown in Figure 1, plasma uric acid level, FBG level, and HOMA‐IR were significantly elevated in all test groups compared to control rats (*p* < .001 for each one). Meanwhile, these parameters were significantly higher in untreated hyperuricemic fructose‐supplemented rats (group III) compared to its corresponding group [untreated hyperuricemic rats (group II)](*p* < .001 for each one). However, such parameters were significantly lowered in l‐Carnitine‐treated hyperuricemic rats (group IV) compared to the corresponding untreated hyperuricemic rats, and in l‐Carnitine‐treated hyperuricemic fructose‐supplemented rats (group V) compared to group III (*p* < .001 for each one). Also, HOMA‐B was significantly elevated in all test groups compared to the control rats (*p* < .001 for each one), as shown in Table 1. On the other hand, nonsignificant changes were observed in plasma insulin levels among the different studied groups, as shown in Table 1.

**Figure 2:**
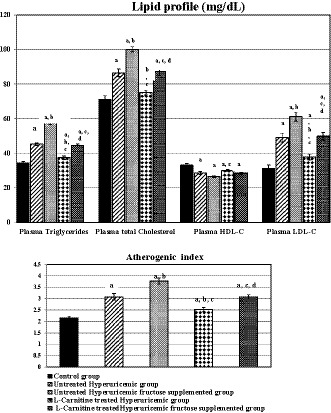
Lipid profile changes in plasma in the different studied groups. Number of rats in each group is 10 rats. (a) Significance by LSD at *p* < .05 compared to control group (I). (b) Significance by LSD at *p* < .05 compared to untreated hyperuricemic group (II). (c) Significance by LSD at *p* < .05 compared to untreated hyperuricemic fructose‐supplemented group (III). (d) Significance by LSD at *p* < .05 compared to l‐Carnitine‐treated hyperuricemic group (IV)

As shown in Figure 2, dyslipidemia was present in hyperuricemic rats and in fructose‐supplemented hyperuricemic rats compared to control ones (*p* < .001 in each one). Fructose supplementation caused significant rises in all lipid profile parameters compared to untreated hyperuricemia group (*p* < .001 in each lipidemic parameter) except plasma HDL‐C level, which was insignificantly changed. l‐Carnitine treatment to either hyperuricemic rats (group IV) or fructose‐supplemented hyperuricemic rats (group V) caused significant improvement of the hyperuricemia‐induced dyslipidemia compared to its respective untreated group, despite having insignificantly changed plasma HDL‐C level.

**Figure 3:**
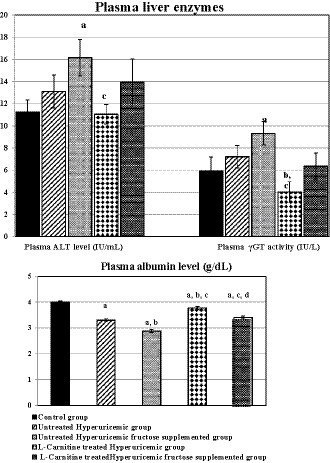
Liver function tests in the different studied groups. Number of rats in each group is 10 rats. (a) Significance at *p* < .05 compared to control group (I). (b) Significance at *p* < .05 compared to untreated hyperuricemic group (II). (c) Significance at *p* < .05 compared to untreated hyperuricemic fructose‐supplemented group (III). (d) Significance at *p* < .05 compared to l‐Carnitine‐treated hyperuricemic group (IV)

Regarding liver functions, as shown in Figure 3, plasma ALT level and plasma γ‐GT activity were insignificantly changed among the different studied groups except in group III, which had significant rises in both liver function tests compared to the control ones (*p* < .05 for both). However, l‐Carnitine‐treated hyperuricemic rats had significantly lowered plasma γ‐GT activity compared to untreated hyperuricemic group (*p* < .02). In addition, plasma albumin levels were significantly reduced in all studied test groups compared to the controls (*p* < .001 in each group except in group IV, *p* < .01), and in group III compared to group II (*p* < .001). On the other hand, l‐Carnitine treatment to either states of hyperuricemia significantly elevated plasma albumin level when each treated group was compared to its respective untreated group (*p* < .001 in each one).

**Figure 4:**
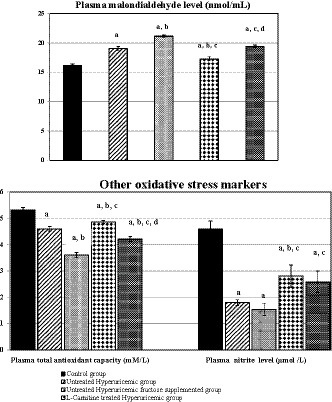
Changes in oxidative stress markers in the different studied groups. Number of rats in each group is 10 rats. (a) Significance by LSD at *p* < .05 compared to control group (I). (b) Significance by LSD at *p* < .05 compared to untreated hyperuricemic group (II). (c) Significance by LSD at *p* < 0.05 compared to untreated hyperuricemic fructose‐supplemented group (III). (d) Significance by LSD at *p* < .05 compared to l‐Carnitine‐treated hyperuricemic group (IV)

Regarding oxidative stress, oxidative stress was evident in all test groups compared to the control group. Concomitant fructose supplementation significantly elevated plasma MDA level and lowered plasma TAC when compared to untreated hyperuricemic group (*p* < .001 for each). Meanwhile, l‐Carnitine treatment to hyperuricemic rats slightly reversed the changes in oxidative stress parameters compared to its respective untreated group, as shown in Figure 4.

When comparing l‐Carnitine‐treated fructose‐supplemented hyperuricemic rats to l‐Carnitine treatment to hyperuricemia alone, there were higher plasma uric acid level, hyperglycemia, higher HOMA‐IR, hypercholesterolemia, hypoalbuminemia, and evident oxidative stress (*p* < .001 in each parameter).

### 3.1 Histopathological results

**Figure 5:**
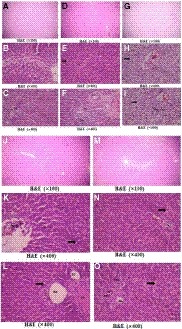
Photomicrographs of H&E‐stained sections of rat livers, (a–c) the control group showing the normal hexagonal or pentagonal liver lobules (a). Hepatocytes were arranged in plates one cell thick, having acidophilic cytoplasm with one or occasionally two spherical nuclei with prominent nucleoli (b). (c) Portal triad containing a branch of hepatic artery (HA), portal vein (PV), and a small bile duct (BD). The phagocytic Kupffer's cells adhered to the endothelial lining of hepatic blood sinusoids (*). (d–f) the hyperuricemic group had slightly disturbed hepatic architecture (arrow in e). (f) Showed features of portal inflammation in the form of dilated congested blood vessels (HA and PV) and mononuclear cellular infiltration (* in f). (g–h) Fructose‐supplemented hyperuricemic group showing extensive hepatic damage, congested central, and portal veins (g). Many necrotic areas with complete destruction of hepatocytes were present as more vacuolated foamy cytoplasm with darkly stained pyknotic nuclei (arrow in h and i), being more at the periphery of the hepatic lobules. (j–k) l‐Carnitine‐treated hyperuricemic group showing nearly normal hepatic architecture, but some hepatocytes were still having vacuolated cytoplasm (arrow) and normal vesicular nuclei (k, l) with little leukocytic infiltration at the portal triad (l). (m‐o) l‐Carnitine‐treated fructose‐supplemented hyperuricemic group showing apparently normal hepatic architecture, despite the presence of more hepatocytes with vacuolated cytoplasm (arrow in n and o) but with normal vesicular nuclei (n) and with little leukocytic infiltration at the portal triad (o)

Light microscopic examination of H&E‐stained sections of rat livers (Figure 5) revealed the characteristic appearance of normal hepatic tissue in the control group (Figure 5a–c). The liver tissue showed the classic hexagonal or pentagonal liver lobules made up of hepatocytes arranged radially around the central vein (Figure 5a). Hepatocytes were arranged in plates with one cell thickness. They have one or occasionally two spherical nuclei with prominent nucleoli; the cytoplasm was acidophilic (Figure 5b). At some of the corners of hepatic lobules, there were small triangular areas of connective tissue containing a branch of hepatic artery, portal vein, and a small bile duct, the portal tract (Figure 5c). The hepatic sinusoids occupied the spaces between the plates of hepatocytes lined by endothelial cells separated from the neighboring plates of hepatocytes by the space of Disse. The phagocytic Kupffer's cells adhered to the endothelial lining of such hepatic sinusoids.

The livers of the hyperuricemic rats were affected in the form of slightly disturbed trabecular structure of the lobules, prominent leukocytic infiltration in addition to mild steatotic changes in the form of many degenerated hepatocytes having vacuolated foamy cytoplasm with darkly stained nuclei (Figure 5d). Some sinusoids were dilated and congested with more Kupffer cells compared to the control group (Figure 5e). Also, features of portal inflammation in the form of dilated congested vessels and mononuclear cellular infiltration were also seen at the portal triads (Figure 5f). Fructose‐supplemented hyperuricemic rats had extensive hepatic damage (Figure 5g). Many necrotic areas with complete destruction of hepatocytes were encountered, in addition to moderate steatotic changes manifested as more vacuolated foamy cytoplasm with darkly stained pyknotic nuclei (Figure 5h). These degenerative changes were most pronounced at the periphery of the hepatic lobules associated with more leukocytic infiltration in the portal triad (Figure 5i).

The livers of l‐Carnitine treatment to either hyperuricemic or fructose‐supplemented hyperuricemic rats showed marked alleviation of the histopathological effects of hyperuricemia that appeared comparable to the controls (Figure 5j and m). However, some hepatocytes were still having vacuolated cytoplasm but with normal vesicular nuclei (Figure 5k), being more in number in l‐Carnitine‐treated fructose‐supplemented hyperuricemic group (Figure 5n), with little leukocytic infiltration at the portal triad (Figure 5l and o).

**Figure 6:**
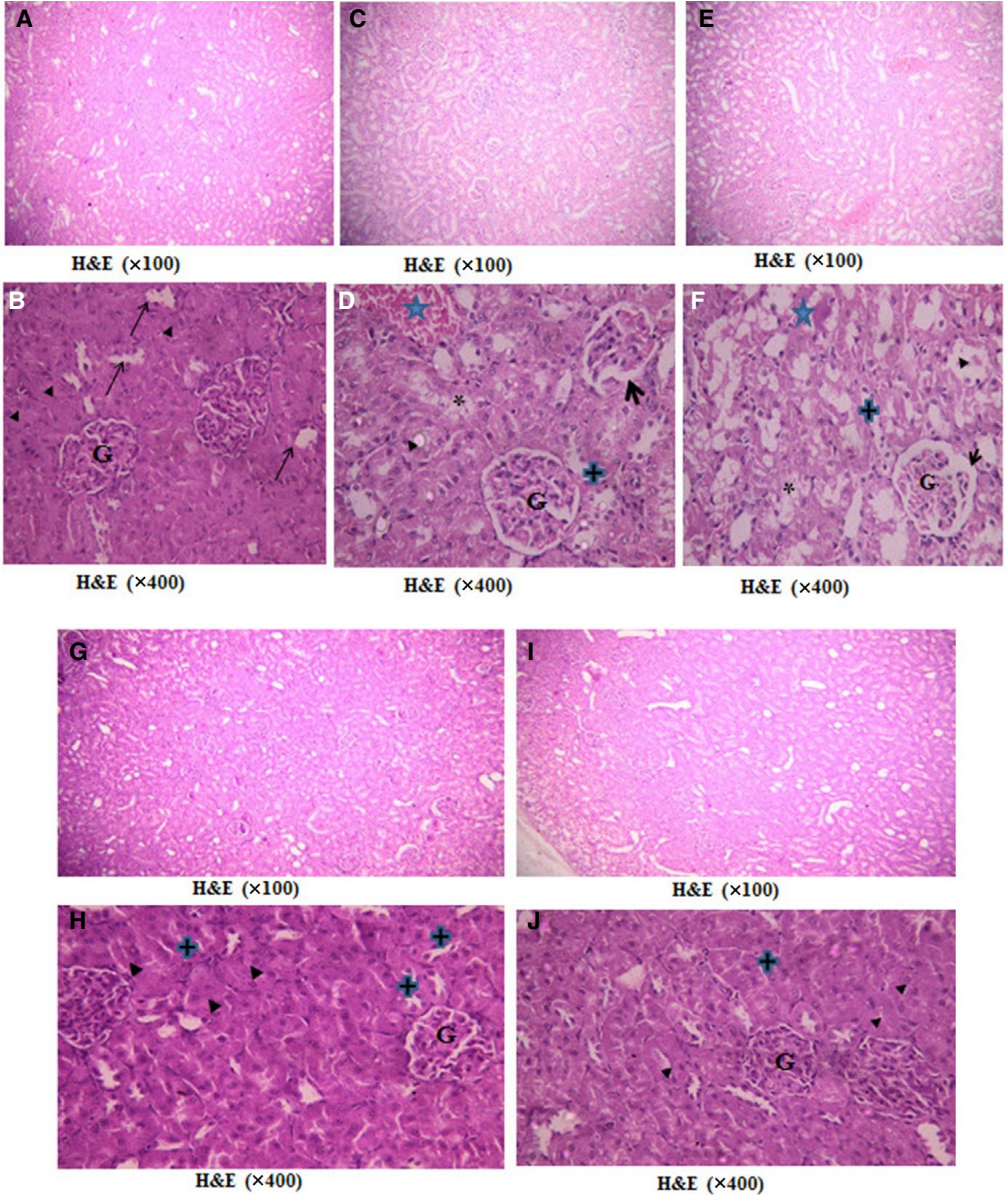
Photomicrographs of H&E‐stained sections of the renal cortex, a and b): the control group showing normal renal architecture formed of renal corpuscles consisting of glomerular capillary tufts (G) surrounded by Bowman's capsule with simple squamous epithelium and narrow Bowman's space. The proximal convoluted tubules (PCT)(▲) were lined with pyramidal epithelium with narrow lumen. The distal convoluted tubules ()were lined with low cuboidal cells with central rounded nuclei and wide lumen. C&D) The hyperuricemic group showing some shrunken glomeruli (G), widened Bowman's space(arrow), congested blood vessels (), acidophilic vacuolated tubular epithelium (*), pale acidophilic cytoplasm, and pyknotic nuclei. E&F) fructose‐supplemented hyperuricemic group had more shrunken glomeruli (G), widened Bowman's space (arrow), more vacuolated cells (*) in PCT(▲) and in DCT (), and acidophilic hyaline casts (). G&H) l‐Carnitine‐treated hyperuricemic rats and l‐Carnitine‐treated fructose‐supplemented hyperuricemic rats (I&J) showed apparently normal glomeruli (G), PCT (▲), and DCT ()

H&E kidney‐stained sections (Figure 6) showed normal structure of the renal cortex and medulla of the control group (Figure 6a). This was markedly affected in the hyperuricemic and fructose‐supplemented hyperuricemic groups (Figure 6c and e). The normal structure was somewhat regained in the l‐Carnitine‐treated hyperuricemic group (Figure 6g), and the l‐Carnitine‐treated fructose‐supplemented hyperuricemic group (Figure 6i).

The renal corpuscles in the different studied groups were demonstrated in Figure 6. In control group (Figure 6a and b), the renal corpuscles were made up of tuft of capillaries (the glomerulus) enclosed inside the Bowman's capsule, that is lined by squamous epithelium. The proximal convoluted tubules (PCT) revealed narrow lumen and were lined by cuboidal cells revealing spherical nuclei and deep eosinophilic cytoplasm. The DCT demonstrated lining cuboidal epithelium and had wider lumen.

H&E kidney‐stained sections of the hyperuricemic rats (Figure 6c and d) revealed the histopathological changes in the form of some shrunken glomeruli, widened Bowman's space, congested blood vessels, acidophilic vacuolated tubular epithelium, pale acidophilic cytoplasm, and pyknotic nuclei. Meanwhile, H&E kidney‐stained sections of fructose‐supplemented hyperuricemic group (Figure 6e and f) revealed more shrunken glomeruli and extensive tubular damage in the form of more vacuolated cells with deeply stained pyknotic nuclei and acidophilic hyaline casts in convoluted tubules.

l‐Carnitine treatment to either hyperuricemic rats (Figure 6g and h) or fructose‐supplemented hyperuricemic rats (Figure 6i and j) showed remarkable regression of such histopathological changes where glomeruli appeared apparently similar to control group, and apparently normal tubules with minimal interstitium in between.

## 4 DISCUSSION

Oxonic acid potassium induces hyperuricemia in rats through inhibiting uricase enzyme resulting in reducing uric acid excretion and higher plasma uric acid levels (Haidari et al., 2012). Similarly, hyperuricemia was evident in untreated hyperuricemic group, herein, while it was aggravated in fructose‐supplemented hyperuricemic rats, in agreement with Nakagawa et al. (2006). This fructose effect could be due to its ability to cause transient intrahepatocellular energy depletion resulting from ATP consumption by fructokinase. This ATP depletion stimulates the degradation of ADP to AMP and inositol with the generation of xanthine and subsequently uric acid (Rosset, Surowska, & Tappy, 2016). Also, fructose was found to stimulate endogenous uric acid synthesis from purine and glycine precursors (Johnson et al., 2013), in addition to reducing uric acid excretion (Chen, Lü, & Yao, 2016).

A novel finding in the current study is the ability of l‐Carnitine to alleviate hyperuricemia, which was not studied before. This decline in uric acid induced by l‐Carnitine supplementation could be due through regulating the energy supply of the cell. Also, l‐Carnitine, being an essential cofactor of carnitine palmitoyltransferase 1 (CPT1), could facilitate fatty acid transport into mitochondria thereby accelerating β‐oxidation. Another possible mechanism is that l‐Carnitine modulates the intramitochondrial acetyl‐CoA/CoA ratio and the pyruvate dehydrogenase complex (Mingorance, Rodríguez‐Rodríguez, Justo, Álvarez de Sotomayor, & Herrera, 2011); however, these mechanisms were not studied herein.

Hyperglycemia and insulin resistance present in hyperuricemic rats, herein, agree with Kodama et al. (2009) and Cicerchi et al. (2014). Similarly, hyperuricemia is a predictor for diabetes mellitus (Lv et al., 2013), and causes hepatic insulin resistance resulted from the associated oxidative stress (Soltani, Rasheed, Kapusta, & Reisin, 2013). The hyperuricemia‐induced oxidative stress could inhibit insulin signaling through the phosphorylation of Akt and insulin receptor substrate‐1 (IRS‐1), and/or lowering phospho‐Akt content in the adipose tissue without changes in total Akt (Zhu et al., 2014).

Oxidative stress was prominent in untreated hyperuricemic rats, herein, similar to Lanaspa et al. (2012) and Lima, Martins‐Santos, and Chaves (2015). Uric acid was found to induce intracellular and mitochondrial oxidative stress through stimulation of NADPH oxidase (Weir, Muir, Walters, & Lees, 2003), and through production of pro‐inflammatory cytokines such as interleukin‐1, interleukin‐6, and tumor necrosis factor‐alpha (Ruggiero et al., 2006). This inflammatory process was present in the histopathological derangement in liver tissues, herein.

Also, the uric acid‐reduced plasma nitrate level, herein, could be mediated by scavenging NO by uric acid itself or by uric acid‐generated oxidants (Gersch et al., 2008).

Therefore, it could be suggested that the coexistent oxidative stress is a causal factor of insulin resistance and hyperglycemia. In support, the leukocytic infiltration of hepatic lobule points to the contributing inflammation in mediating such oxidative stress.

On the other hand, simultaneous fructose supplementation with induction of hyperuricemia, herein, caused more obvious hyperglycemic effect and insulin resistance in accordance to Tapia et al. (2013). Such effects could be attributed to either fructose, or its interaction with uric acid or more prominent oxidative stress (Zhang, Jiao, & Kong, 2017).

In the current study, cotreatment of l‐Carnitine with hyperuricemia induction ameliorated such carbohydrate metabolic derangement, despite not altering the insulin level compared to the untreated hyperuricemic rats, in line with Samimi et al. (2016). Such protective effects of l‐Carnitine could be mediated by preventing the rise of serine phosphorylation of IRS‐1, which negatively regulates insulin signaling (Kon et al., 2017). Also, l‐Carnitine could increase the efflux of acyl and acetyl groups out of the cells into the plasma, reducing the accumulation of these intermediate products of β‐oxidation (Chapela, Kriguer, Fernández, & Stella, 2009), improving insulin resistance (Zhang, Keung, Samokhvalov, Wang, & Lopaschuk, 2010).

Also, l‐Carnitine supplementation to fructose‐supplemented hyperuricemic rats improved the hyperglycemia and insulin resistance, similar to Rajasekar, Ravichandran, and Anuradha (2006). It is noted that l‐Carnitine was more efficient in ameliorating hyperuricemia‐induced insulin resistance in the state of hyperuricemia alone than in fructose‐supplemented hyperuricemic rats, herein.

In addition, dyslipidemia was found in hyperuricemic rats, herein, in agreement with Chen et al. (2007) and Keenan et al. (2012). This dyslipidemia was associated with mild hepatic steatosis, could partly be explained by the ability of uric acid to inhibit adenosine monophosphate‐activated kinase (AMPK) responsible for fatty acid oxidation, and ATP generation (Lanaspa et al., 2012). Another possible mechanism is direct stimulation of hepatic lipogenesis by uric acid through inducing mitochondrial oxidative stress (Lanaspa et al., 2012). In support, uric acid was positively correlated with either LDL‐C or atherogenic index, and negatively correlated with HDL‐C in hyperuricemic rats (Chen et al., 2007).

Meanwhile, combined fructose‐supplemented hyperuricemic rats, herein, exhibited more dyslipidemic effects and extensive liver injury than hyperuricemia alone, in line with Nakagawa et al. (2006) and El‐Kafoury, Abdel Rhman, and Salah El Din (2011). Dyslipidemia and moderate hepatic steatosis in these rats, herein, could be explained by the ability of high fructose to cause hepatic triglycerides accumulation (Tapia et al., 2013), and induce liver injury in mice (Han, Li, Huang, & Yang, 2016), through inducing a defect in the antioxidant defence mechanisms and excessive ROS production (Busserolles, Gueux, Rock, Mazur, & Rayssiguier, 2002), thereby causing excessive lipid peroxidation (Joyeux‐Faure, Rossini, Ribuot, & Faure, 2006).

Such metabolic and hepatic effects of combined fructose administration with hyperuricemia could be, also, explained by excessive fructose‐induced uric acid synthesis and by the lipogenic effect of fructose. In hepatocytes, uric acid could upregulate fructokinase expression through activating the transcription factor carbohydrate responsive element‐binding protein (ChREBP), amplifying the lipogenic effects of fructose (Lanaspa et al., 2012). Thus, it could be suggested that fructose supplementation, herein, accentuated the detrimental effects of hyperuricemia through enhancing hepatic triglyceride synthesis causing a greater need for NADPH and energy consumption, causing higher uric acid production (de Oliveira & Burini, 2012).

The lipid‐lowering effects of l‐Carnitine supplementation, herein, similar to Malaguarnera et al. (2009), could be through carrying long‐chain fatty acids across the inner mitochondrial membrane for β‐oxidation and ATP production (Flanagan et al., 2010). l‐Carnitine, also, lowered plasma triglyceride levels by improving fat utilization (Alipour, Barzegar, Panahi, Safaeian, & Es.haghi, M., 2014). In addition, l‐Carnitine limited the associated hepatic inflammation possibly by upregulating the mitochondrial β‐oxidation and redox system, and reducing IL‐1 and TNF‐α levels in liver (Ishikawa et al., 2014).

These lipolytic and hepatoprotective effects of l‐Carnitine could be, also, mediated by attenuating lipotoxicity, thereby limiting the metabolic abnormalities and hepatocyte damage (Jun et al., 2011). As excess uric acid could induce dyslipidemia through inhibition of fatty acid oxidation, and triggering inflammation and oxidative stress, l‐Carnitine could interfere with such effects through its antisteatotic, anti‐inflammatory, and antioxidant activities, evidenced herein by reducing the hepatic leukocytic infiltration, causing higher TAC and reduced plasma MDA level (Ishikawa et al., 2014; Kolodziejczyk et al., 2011; Lee, Lin, Lin, & Lin, 2016; Ribas et al., 2014).

Regarding liver functions and structure, hyperuricemia caused mild liver cell injury and hypoalbuminemia compared to the controls, despite causing insignificant changes in plasma ALT level and γ‐GT activity. These findings disagree with Afzali, Weiss, Boyko, and Ioannou (2010), who found that hyperuricemia was associated with chronic liver disease in humans. This discrepancy between the current study and other studies could be related to the duration of hyperuricemia.

The prominent inflammatory process in the liver of hyperuricemic rats could be caused by oxidative stress (Yang et al., 2016). The degree of hepatic architecture derangement, herein, was more evident in fructose‐supplemented hyperemic rats than in untreated hyperuricemic rats. This denotes that the liver cell injury induced by hyperuricemia alone was not sufficient to elevate the liver enzymes in plasma.

Meanwhile, hyperuricemic rats had shrunken renal glomeruli and tubular damage, which may be due to the prominent oxidative stress in these rats. Thus, the hyperuricemia‐induced hypoalbuminemia, herein, could be explained by such renal damage rather than the hepatic changes. In line, hyperuricemia was associated with inflammation, oxidative stress, renal hyperfiltration, proteinuria, and chronic kidney disease (Chaudhary, Malhotra, Sowers, & Aroor, 2013).

On the other hand, fructose‐supplemented hyperuricemic rats exhibited impaired liver function, and had extensive liver cell damage, similar to Lanaspa et al. (2012). They attributed such changes to the aggravating effects of fructose on hyperuricemia together with oxidative stress. It could be suggested that the fructose elevated the uric acid to threefolds of control values, herein, and caused nonalcoholic steatohepatitis. Also, the hypoalbuminemia could be, also, explained by the observed renal structural derangement in fructose‐supplemented hyperuricemic rats.

Meanwhile, l‐Carnitine administration to either states of hyperuricemia limited hepatic damage, ameliorated inflammatory cell infiltrate, and reduced liver enzymes in plasma. These hepatoprotective effects could be attributed to protection of hepatocytes against oxidative stress‐induced cell damage by l‐Carnitine (Li, Wang, Luan, Kang, & Wang, 2012).

Regarding anthropometry, hyperuricemia caused insignificant changes in the studied anthropometric measures compared to the controls, which contradict with the hyperuricemia‐induced dyslipidemia, and the stimulated lipogenesis induced by higher uric acid. These findings could be explained by hyperuricemia‐induced fatty liver independent of obesity. In line, hyperuricemia was found to be associated with NAFLD in hemodialysis subjects with BMI below 20 (Malaguarnera et al., 2009).

On the contrary, coadministration of fructose with hyperuricemia induction, herein, caused adiposity. Fructose was suggested to cause such weight gain due to the defective ability of fructose in stimulating leptin secretion, without subsequent satiety response (Teff et al., 2004). Fructose, also, encourages food intake through stimulation of either dopamine in the limbic system (Bernal et al., 2008), or by hepatic ATP depletion (Bawden et al., 2012).

Fully unexpected, l‐Carnitine supplementation together with hyperuricemia caused adiposity, herein, in discordance with Jang et al. (2014). Other studies found that carnitine supplementation did not assist in weight loss (Benvenga, 2005;Brass, 2004). Thus, the weight gain in l‐Carnitine‐treated hyperuricemic rats was unreasonable and hard to be explained herein. However, the anti‐adiposity effects of l‐Carnitine in fructose‐supplemented hyperuricemic rats could be attributed to its lipolytic and antioxidant effects according to Flanagan et al. (2010) and Derosa et al. (2011), in addition to eliciting more energy expenditure (Kim, Pan, Lee, & Kim, 2015).

This study has a novel finding which is the ability of l‐Carnitine to strongly combat against hyperuricemia‐induced metabolic changes, as l‐Carnitine was not previously used for the treatment of hyperuricemia except Loots et al. (2004), who revealed that l‐Carnitine limited oxidative stress markers and uric acid production. Meanwhile, l‐Carnitine supplementation, herein, limited the aggravating effects of fructose on such detrimental hyperuricemic effects. However, the effects of l‐Carnitine supplementation to either normal rats or fructose‐supplemented rats having normal plasma uric acid levels were not studied herein, which is a limitation of the current study.

It could be concluded that hyperuricemia caused deleterious metabolic and hepatic effects. Artificial fructose supplementation aggravated the picture of hyperuricemia, and showed more deleterious metabolic and hepatic effects. l‐ Carnitine, through its anti‐inflammatory, antisteatotic, and antioxidant effects, was efficient in ameliorating such effects of hyperuricemia and/or combined excess fructose intake and hyperuricemia.

## CONFLICT OF INTEREST

None declared.
